# The Influence of Leader Bottom-Line Mentality on Miners’ Safety Behavior: A Moderated Parallel Mediation Model Based on the Dual-System Theory

**DOI:** 10.3390/ijerph191811791

**Published:** 2022-09-19

**Authors:** Lixia Niu, Wende Xia, Yafan Qiao

**Affiliations:** School of Business and Management, Liaoning Technical University, Huludao 125100, China

**Keywords:** leader bottom-line mentality, safety behavior, emotional exhaustion, safety consciousness, Chinese traditionality, dual-system theory

## Abstract

As a high-risk industry that is always struggling with unsafe factors, coal mine enterprises must prioritize safety in their operation and management, but there are still some short-sighted coal mine managers who choose to leave safety behind in the desperate pursuit of financial benefits, resulting in coal mine accidents from time to time. Unfortunately, this leadership style, known as leader bottom-line mentality, has not yet received sufficient attention in the safety field. Based on dual-system theory, this study aimed to explore the mediating role of emotional exhaustion and safety consciousness between leader bottom-line mentality and miners’ safety behavior, as well as the moderating role of Chinese traditionality. Using a sample of 422 frontline miners in China, the results of the data analysis showed that emotional exhaustion and safety consciousness played parallel mediating roles between leader bottom-line mentality and miners’ safety behavior, and Chinese traditionality moderated the effect of leader bottom-line mentality: the higher the Chinese traditionality, the weaker the mediating effect of emotional exhaustion and the stronger the mediating effect of safety consciousness. Present research explains the mechanisms and boundaries of the influence of leader bottom-line mentality on miners’ safety behavior, contributing to the emerging literature on safety management and bottom-line mentality.

## 1. Introduction

Although China has always been committing to the pursuit of clean and safe energy, given coal-rich, oil-poor, and gas-poor national conditions, China will not be able to change its coal-based energy structure in a short time. Coal remains the main source of energy in China, providing more than 70% of the country’s energy consumption [1,2]. The coal mining industry has always been recognized as a high-risk industry worldwide [3,4]. In recent years, as the Chinese government pays more attention to coal mine safety, with increased investment in coal mine safety and the upgrading of mining equipment, the total number of coal mine safety accidents and casualties has decreased year by year [5]. Human-caused accidents in coal mines still occur from time to time and are one of the most important problems of coal mine safety in China, and China’s coal mine safety performance also lags behind that of fellow major coal producers such as Australia and India [1,6]. In the analysis of the causes of accidents in coal mines, many studies have found that human error is one of the major causes of coal mine accidents, and it mainly stems from leaders’ mismanagement and miners’ unsafe behavior [2,7,8]. Therefore, correcting leaders’ mismanagement and reducing miners’ unsafe behaviors are essential to improving the overall safety performance of coal mines.

It is well known that employees’ unsafe behavior is a direct contributor to most accidents [9]. The management failure of top coal mine leaders is considered to be the most unsafe behavior and difficult to correct [2], and is often the key situational factor that leads to unsafe employee behavior [10]. According to social information processing theory, individuals construct their perceptions and attitudes by processing social cues in the workplace, and these perceptions and attitudes in turn influence their behavior [11]. As the distributor of information and allocator of resources within the organization, leaders have a key role in influencing employees’ safety behaviors [11,12]. Previous researchers have mostly explored the relationship between constructive leadership and workplace safety behaviors based on a positive organizational behavior perspective. For example, studies have shown that safety leadership [13], temporal leadership [14], and spiritual leadership [15] all positively promote employee safety behaviors, but lack sufficient attention to the destructive effects of dysfunctional leadership [12,16], and only a few studies have focused on the effect of abusive management [17] and passive leadership [18] on subordinates’ safety behaviors. In addition, in Chinese organizational contexts with high power distance, especially for coal mining companies, the relatively closed environment and hierarchical organizational levels are more likely to foster negative leadership [19]. Therefore, it is necessary to further investigate the influence of negative leadership on miners’ safety behaviors in the Chinese context.

As a high-risk industry that is always struggling with unsafe factors, coal enterprises must give top priority to safety in their operation and management [2]. Unfortunately, however, there are still some coal mining enterprise managers who do not strictly implement safety responsibilities and ensure adequate safety investment for short-term economic benefits, resulting in accidents from time to time [20]. This leadership style, which ignores competing organizational priorities for the sake of organizational economic benefits, is known as leader bottom-line mentality (LBLM) [21]. In today’s intense and ever-changing business environment, the bottom-line mentality is seen as a double-edged leadership that lacks vision but is very practical [22,23]. However, when this profit-oriented leadership is present in the safety workplace, it may lead to disastrous results. Fruhen et al. [24] argued that a key role for leaders is to support employees to make sense of organizational priorities; specifically, in the context of safety-critical workplace, leaders are expected to put safety as organizational priority. In contrast, the LBLM usually chooses to consider financial performance as the bottom-line outcome of the organization, which is likely to lead to lower performance in other competing matters (e.g., creativity, safety performance) [25]. Unfortunately, current literature on LBLM has focused on general business organizational situations and has not attracted much attention to its destructive outcomes in the safety workplace. Therefore, in order to clarify the destructiveness of the LBLM to the safety-critical workplace, and respond to the call to examine the impact of other leadership styles on employee safety behavior and to explore the effect of the LBLM in other industry contexts [26,27], this study will further explore the potential negative impact and mechanism of LBLM on employee safety behavior.

To better understand this mechanism, this study draws on the dual-system theory, which posits that individuals have two relatively independent cognitive systems: a fast, automated, unconscious system 1 and a slow, controlled, conscious system 2 [28]. Based on this theory, this study proposes that LBLM affects miners’ safety behaviors by influencing both cognitive system 1 and system 2. Specifically, on the one hand, LBLM fosters a vicious competitive climate within the organization and demonstrates disregard for organizational safety [21,29], which leads to employees’ concern about their job safety and life safety [30,31]. Such a negative leadership style can lead to miners’ emotional exhaustion [32], which makes employees’ system 2 paralyzed and unable to use correct coping behaviors when facing safety decision-makings [12] The LBLM instills employees with financial benefits over safety, which subconsciously undermines their perceptions of safety. This can lead to a decrease in miners’ safety consciousness and contamination of safety-specific system 1, making it difficult for miners to effectively identify safety hazards in the workplace [33]. Given that little previous literature has investigated LBLM in the safety workplace, based on the dual-system theory, the present study explores the mechanism of LBLM on miners’ safety behavior from the dual-path perspective of employee emotional exhaustion and safety consciousness.

In addition, the boundary that influences the role of LBLM still needs to be further explored. Previous studies have noted that there may be significant differences in the response of individuals with different cultural value orientations when faced with LBLM, but the current focus on subordinate cultural value orientations remains insufficient [27]. Indeed, individuals are not passive receivers of information, their subjective interpretations of information play an important role in the process of social information processing. Individuals with different cultural values may interpret social information differently in the same context [34]. Therefore, this study selects Chinese traditionality as a moderating variable and proposes that Chinese traditionality, the extent to which an individual endorses the traditional hierarchical relationships prescribed by Confucian social ethics (p. 717) [35], which may be an important boundary for the LBLM to influence subordinates’ cognition and behavior.

**Figure 1:**
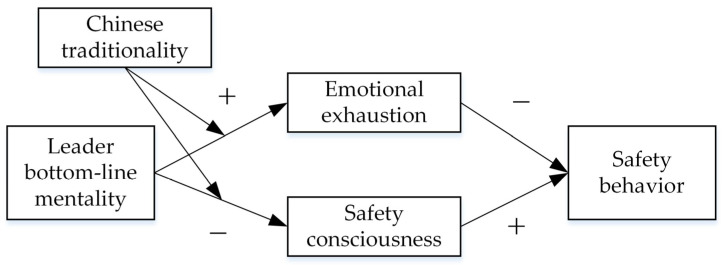
Theoretical model.

This study aims to make the following theoretical contributions: first, this study examines the effects of LBLM on miners’ safety behaviors, responding to previous literature calls to explore the effects of LBLM in different industries and expanding the results of LBLM [27]. It is further proposed that when the expected outcome is contrary to the bottom-line outcome, the LBLM will completely change into a dysfunctional leadership style. Second, based on dual-system theory, this study proposes the mechanism of LBLM in influencing subordinates’ safety behaviors, further enriching the literature related to dual-system theory and safety behavior. Previous literature suggests that individuals’ safety behaviors are jointly determined by the automatic cognitive system 1 and the controlled cognitive system 2, and integrating consideration of controlled and automatic cognitive processes helps to better explain safety behaviors in the workplace [12,33,36,37]. Based on above, this study further proposes that LBLM can not only lead to miners’ emotional exhaustion to destroy their cognitive system 2 so that they cannot practice safety behaviors, but also reduce subordinates’ safety consciousness to contaminate their cognitive system 1 so that they gradually lose the correct perception of safety. Finally, this study proposes Chinese traditionality as a boundary for the LBLM to influence employees’ safety cognition and safety behaviors. Regardless of the cultural values held by individuals in a safe workplace, the LBLM will impair individuals’ safety behaviors, only the path of its effects may differ. Figure 1 shows the theoretical model for this study.

## 2. Literature Review and Hypothesis Development

### 2.1. Leader Bottom-Line Mentality and Safety Behavior

Based on the form of task performance, Griffin and Neal [38] defined safety behaviors as safety compliance and safety participation. Safety compliance behaviors are basic activities that individuals perform to maintain their safety, such as wearing safety equipment as required. Safety participation behaviors are proactive behaviors that employees engage in spontaneously to help maintain a safe organizational environment, such as actively participating in safety training activities [9,14]. The active practice of safety behaviors by employees can reduce the occurrence of accidents and injuries in the workplace and is key to improving organizational safety performance. Therefore, it is important to explore the contributors, inhibitors, and formation mechanisms of employee safety behaviors.

The LBLM is a dysfunctional leadership in which leaders focus only on bottom-line outcomes (usually financial performance) and ignore other competing priorities [21,25,39]. The bottom-line mentality is characterized by one organizational factor that is viewed as extremely important, while all other factors are relatively ignored [39]. Leaders with a high bottom-line mentality focus on achieving the company’s bottom-line results and neglect the well-being of their employees [22,40]. In recent years, LBLM has attracted much attention due to its dysfunctional nature and destructive impact on long-term organizational development [25,40,41]. For example, Greenbaum et al.’s findings suggested that LBLM shapes employees’ bottom-line mentality through social learning effects, which in turn leads to employees’ social undermining behaviors [21]. Quade et al. [40] suggested that LBLM leads to low-quality social relationships that make employees’ task performance decline. In addition, other studies have found that LBLM inhibits team creativity [42] and leads to work-family conflict [23]. It has also been suggested that LBLM is a practical management style that promotes employees’ perceived obligation to the bottom-line and mental focus on their work leading to positive performance [27]. However, the present study argues that such positive outcomes are predicated on the assumption that the leader’s bottom-line outcomes are consistent with expected performance. For example, the pursuit of financial performance bottom-line leads to positive financial outcomes but may impair performance in other competing matters (e.g., creativity, safety performance, etc.) [42]. Therefore, this study argues that when this leadership style of pursuing financial performance at all costs appears in coal mining enterprises, it may inhibit safety performance.

Based on the self-determination theory, this study believes that the LBLM will influence the employees’ safe behavior by affecting their controlled motivation and autonomous motivation [43]. First of all, in pursuit of higher economic benefits, LBLMs will use their power to force employees to work in unsafe working conditions or uncomfortable physical conditions, force them to engage in unsafe operational behaviors. Second, LBLM will stimulate employees’ extrinsic motivation through rewards and punishments. When employees’ performance contributes to bottom-line results, they will be rewarded, otherwise, they will be punished. In this context, employees will make extra effort to achieve desired outcomes and avoid unfavorable outcomes, even though the behavior may not comply with safety regulations [21]. In addition, LBLM will also affects miners’ autonomous motivation. In a high-reliability organization, in order to ensure the safety of employees and the organization, leaders will strictly supervise employees to fulfill the safety norms stipulated by the organization [44]. In fact, wearing personal protective equipment and going through onerous safety procedures at all times may not be a pleasant experience for some employees, and they may not instinctively want to do so. Especially under the management of the LBLM, when employees are in such an organization that pays attention to economic effects and relatively ignores safety, employees are not bound by leaders and strict safety norms, employees may breed more paralysis, fluke, and convenient psychologies [2]. Accordingly, this study proposes the following hypothesis:

**Hypothesis** **1** **(H1).** *LBLM has a negative effect on miners’ safety behavior.*

### 2.2. Dual-System Model and Safety Behavior

In previous studies, the dual-system theory has provided the theoretical basis for explaining the formation mechanism of safety behavior [12,33,36]. According to dual-system theory, individuals possess two relatively independent cognitive systems: a fast, automated, unconscious system 1 and a slow, controlled, conscious system 2 [28]. The significant difference between system 1 and system 2 is that the activation of system 1 does not require the individual’s working memory resources, whereas the activation of system 2 requires these resources, which determines that an individual’s system 2 is limited by the individual’s cognitive resources [45]. In particular, when employees’ cognitive resources are depleted, the individual’s system 2 will not function properly, making it impossible for employees to make rational and correct judgments during the work process, which in turn leads to a decrease in the level of safety participation and safety compliance [33]. In addition, most studies have concluded that when an individual’s cognitive system 2 is not functioning properly, system 1 can act as a secondary source for responding that can spontaneously and effortlessly support employees’ safety behaviors [12,33]. Chen et al. [36] argued that employees in High Reliability Organizations also exhibit higher levels of safety compliance behavior when they experience a depletion of self-control resources (i.e., system 2 failure) based on strong safe implicit beliefs that have developed over time.

However, it is not only the individual’s system 2 that can be damaged, but the individual’s system 1 may also be at risk of being contaminated. In fact, system 1 is not as reliable as expected, and when individuals store false implicit beliefs (e.g., believing that it is right to complete work on time while violating safety rules), it will lead to wrong decisions [33]. Further, according to dual-system theory, the normal operation of cognitive system 2 is often based on cognitive system 1 [45]. Therefore, the failure of individual system 1 may be more harmful than that of system 2, and it is also important to explore the circumstances in which system 1 is contaminated. In addition, different from other negative leadership commonly seen in workplaces (e.g., abusive supervision), LBLM not only leads to severe depletion of employees’ emotional resources, making the cognitive system 2 impaired and unable to make proper safety decisions, but also may indoctrinate employees with wrong safety concepts, as a result, employees distort the correct cognition of safety behavior due to the degeneration of cognitive system 1. To sum up, based on the dual-system theory, this study explores the mechanism of LBLM influencing employees’ safety behaviors from the dual path perspective of impaired cognitive system 2 and contaminated cognitive system 1.

### 2.3. Mediating Role of Emotional Exhaustion

Emotional exhaustion refers to feelings of being emotionally overwhelmed and depleted [46,47]. Existing research suggests that negative leadership styles as stressors in the workplace are often direct antecedents of subordinates’ emotional exhaustion. For example, Elsaied [48] suggested that exploitative leadership leads to emotional exhaustion by exerting work pressure on employees and limiting their career development. Wan et al. [32] study confirmed that LBLM leads to a significant loss of emotional resources and a state of emotional exhaustion. Emotional exhaustion in individuals often leads to numerous negative outcomes. It has been shown that emotional exhaustion is directly related to employees’ deviant behavior [49], unsafety behavior [50], and knowledge-hiding behavior [51]. According to dual-system theory, the emotional exhaustion state implies that employees lack sufficient resources for self-control, which can disable the individual cognitive system 2 and lead to employees exhibiting lower levels of safety behaviors [12]. Therefore, given the established relationships between LBLM and emotional exhaustion, emotional exhaustion, and safety behaviors in the existing literature [12,32], based on dual-system theory, this section focuses on the mediating role of employee emotional exhaustion between LBLM and employees’ safety behaviors.

Firstly, leaders with a bottom-line mentality set high performance goals for employees in order to ensure the financial performance of the organization, making it difficult for employees to balance safety and task performance. Excessive work stress and anger at leaders for belittling safety make employees vulnerable to falling into a state of emotional exhaustion [52,53]. Secondly, in order to better achieve their bottom-line goals, leaders may force employees to work for longer and more intensely, making it difficult for employees to recover their emotional resources [29,54]. Thirdly, the LBLM of overemphasis on financial performance is often accompanied by a decrease in investment in organizational safety. The deterioration of workplace safety and the leader’s relatively indifferent attitude toward safety can cause employees to worry about the safety of their work environment, and the prolonged state of concern for their safety can lead to emotional exhaustion [5,31]. Finally, the LBLM creates a competitive climate in the organization, and this vicious competition makes it difficult to establish good interpersonal relationships among employees. Leaders who neglect the well-being of employees and colleagues who are indifferent to interpersonal relationships leave employees without the necessary emotional resources to replenish them, which further leads to emotional exhaustion [21,29].

According to dual-system theory, when individuals have sufficient resources for self-control, individuals’ system 2 will enable them to avoid incorrect or socially undesirable behaviors through rational decision-making. However, LBLM can lead employees to emotional exhaustion, leaving them without sufficient cognitive resources to keep system 2 functioning properly, which in turn reduces employee safety behavior [12,50]. Specifically, impairment of system 2 can lead to a lack of adequate self-control and rationality when employees are faced with safety behavior decisions [12]. This can lead to the development of a fluke, and convenient mentality [2], and employees are more inclined to disobey safety rules and choose to take shortcuts in order to save resources, resulting in lower levels of safety compliance. In addition, it has been shown that employees’ extra-role behaviors, such as employee helping behaviors, can also lead to emotional exhaustion [55]. Therefore, employees who are already emotionally depleted may show indifference to people and events in the work environment and reduce their extra-role behaviors, such as safety participation, in order to prevent further deterioration of emotional exhaustion and reduce the consumption of cognitive resources [51]. In summary, the following hypothesis is proposed in this study:

**Hypothesis** **2** **(H2).** *Emotional exhaustion mediates the negative effect of LBLM on miners’ safety behavior.*

### 2.4. Mediating Role of Safety Consciousness

Safety consciousness refers to individuals’ own awareness of safety issues [56]. At the cognitive level, safety consciousness is the individual’s mental perception of what safety is and what safety behaviors are. At the behavioral level, safety consciousness promotes individuals to engage in safety-specific behaviors [57]. Employees’ safety consciousness is malleable, which may be closely related to the environment in which they work. For example, Seibert [58] found that employees can acquire safety consciousness through organizational safety-centered policies and training. However, the LBLM emphasizes the organization’s financial performance as the centerpiece, which conveys the misconception that financial performance takes precedence over safety in the workplace and is clearly detrimental to the development of employee safety consciousness. The present study argues that the decrease in safety consciousness can be viewed as a contamination effect of the LBLM on subordinates’ cognitive systems 1. Therefore, based on dual-system theory, this study argues that LBLM leads to lower miners’ safety consciousness, which makes miner’s cognitive system 1 deteriorate and thus exhibit lower levels of safety behavior.

As the face of the organization, the leader holds the power to distribute information and allocate resources to the organization. According to social information processing theory, individuals construct their perceptions and attitudes by processing social cues in the workplace, and these perceptions and attitudes will in turn influence their behavior [11]. Therefore, the social information revealed by leaders often becomes the basis for employees’ perceptions and practices in the workplace. It has been shown that both safety-specific transformational leadership and ethical leadership, which focus on safety issues within the organization and safety training for employees, significantly contribute to subordinates’ safety consciousness [57,59]. However, the LBLM that prioritizes financial performance is clearly not conducive to the development of subordinates’ consciousness. Specifically, firstly, leaders with a bottom-line mentality center on the financial interests of the organization, which conveys the social information to employees that financial performance takes precedence over safety performance, and in such an environment, employees may subconsciously internalize the leader’s mentality, making them lack proper perceptions of safety and leading to a decline in safety consciousness [21]. For example, Zhang et al. [60] suggested that LBLM can weaken employees’ self-regulatory mechanisms and cause employees to cognitively reconstruct pro-organizational unethical behaviors into reasonable behaviors. Similarly, the LBLM instills employees with the wrong safety perception, which may cause employees to cognitively reconstruct dangerous shortcut behaviors as efficient and correct ways of working. Secondly, LBLM urges employees to strive for bottom-line outcomes through rewards and punishments [21,61]. Employees may actively internalize the leader’s misguided idea of prioritizing economic benefits in order to maximize their interests, leading to a diminished sense of safety. Finally, when leaders prioritize financial benefits in their organizations, they are likely to reduce the organization’s safety investments (e.g., safety training, safety drills) to reduce costs [5]. When an organization lacks access to safety knowledge, employees with little safety knowledge do not know what safety behaviors are and how they should be performed.

According to dual-system theory, system 1 serves as a cognitive basis for the rational analysis and decision-making process of system 2, and a second response mechanism after the failure of system 2, which can effortlessly guide employees’ safety behaviors [12,33]. However, the individual’s system 1 is not always reliable, and it stems from the individual’s implicit beliefs about safety that have been formed over time, and the implicit beliefs formed over time by employees who lack safety consciousness may be incomplete or even wrong, which can cause employees’ safety behaviors at serious risk [33]. On the one hand, even when individuals are in a state of adequate cognitive resources and system 2 is functioning normally, the lack of safety consciousness can lead to employees’ inability to make correct judgments when faced with safety decision-making due to the lack of sufficient safety information. On the other hand, when an individual’s system 2 does not function properly due to the lack of cognitive resources, system 1 will take over the control of the behavior, and individuals with low safety consciousness store false safety implicit beliefs over time (e.g., it is reasonable to break rules in order to complete work tasks), which can lead employees to engage in unsafe behaviors, either intentionally or unintentionally. In summary, the following hypothesis is proposed in this study:

**Hypothesis** **3** **(H3).** *Safety consciousness mediates the negative influence of LBLM on miners’ safety behavior.*

### 2.5. Moderating Role of Chinese Traditionality

Chinese traditionality refers to “the extent to which an individual endorses the traditional hierarchical role relationships prescribed by Confucian social ethics” [35]. Individuals with high traditionality regard hierarchical role relationships and social expectations as the code of conduct, emphasizing the fulfillment of unilateral obligations to authority figures without paying much attention to the attitudes and behaviors that authority figures treat them [35,62]. Especially in the field of organizational management, individuals with high Chinese traditionality tend to obey and be loyal to the leader unconditionally [63]. In contrast, individuals with low traditionality tend to act based on the inducement-contribution balance with the leader and value the fulfillment of mutual obligations between the leader and subordinates [35]. Individuals with different levels of Chinese traditionality may have different interpretations when they face the same social information [64]. Therefore, the present study suggests that there may be differences in which individuals with different Chinese traditionality have contaminated system 1 and impaired system 2 when faced with LBLM.

On the one hand, Chinese traditionality moderates the positive influence of the LBLM on miners’ emotional exhaustion. Specifically, high Chinese traditionality adheres to the role concept of superiority and inferiority and follows the authority of the leader, and even if the leader neglects the safety and well-being of the employees, the employees will believe that the leader has a reason for doing so, so when faced with a leader who has the bottom-line mentality, high Chinese traditionality employees have a weaker sense of psychological contract violation and negative emotions, and emotional exhaustion is mitigated [64,65], while for employees with low Chinese traditionality, who uphold the principle of equal social exchange and emphasize the fulfillment of two-way obligations between leaders and subordinates, there is a more violent reaction to leaders’ disregard for employee’s safety [34]. However, due to the leader’s authority and the huge potential cost of resigning in the COVID-19 period, miners can only suppress their dissatisfaction with the leader, which causes them to experience higher levels of emotional exhaustion.

On the other hand, we propose that Chinese traditionality moderates the negative impact of the LBLM on miners’ safety consciousness. Employees with high Chinese traditionality tend to unconditionally obey and be loyal to their leaders when faced with the misconception that leaders’ financial interests take precedence over safety, and are less likely to question their leaders’ attitudes and behaviors, and therefore internalize this misconception more quickly and proactively, reducing employees’ safety consciousness more significant [63,66], while miners with low traditionality will rationalize the social messages conveyed by authority figures and resist the wrong ideas conveyed by leaders within the organization, allowing the negative effect of the LBLM on safety consciousness to be mitigated. In summary, this study proposes the following hypotheses:

**Hypothesis** **4a** **(H4a).** *Chinese traditionality moderates the positive relationship between LBLM and miners’ emotional exhaustion. The higher the employee’s Chinese traditionality, the weaker the positive effect of the LBLM on miners’ emotional exhaustion.*

**Hypothesis** **4b** **(H4b).** *Chinese traditionality moderates the negative relationship between LBLM and miners’ safety consciousness. The higher the Chinese traditionality of employees, the stronger the negative effect of LBLM on miners’ safety consciousness.*

So far, based on the dual-system theory, this study proposes that LBLM leads to subordinates’ emotional exhaustion (system 2 is impaired) and reduces subordinates’ safety consciousness (system 1 is contaminated), which in turn inhibits employees’ safety behaviors. Based on this, this study further suggests that Chinese traditionality may moderate the mediating role of emotional exhaustion and safety consciousness between the LBLM and subordinates’ safety behaviors. Specifically, when confronted with LBLM, individuals with high Chinese traditionality tend to obey the leader’s orders and internalize the information and ideas conveyed by the leader, and are less likely to have strong negative emotions due to the way the leader treats them. Therefore, such individuals will experience lower emotional exhaustion and a decline in safety behavior due to the damaged cognitive system 2 being alleviated, while for individuals with low traditionality, individuals will perceive that the leader does not fulfilling the basic psychological contract of safety and lacks attention to employees’ safety and well-being, and will experience higher levels of emotional exhaustion, further amplifying the inhibitory effect on safety behaviors.

On the other hand, the blind acceptance of the leader’s erroneous ideas by individuals with high traditionality will lead to a more serious decline in their safety consciousness, and the cognitive system 1 is contaminated to a deeper degree, making the employees’ safety behavior further decline. However, individuals with low traditionality will question the leader’s faulty thinking and management style, and refuse to internalize the misconception that financial interests take precedence over safety, mitigating the inhibitory effect of the LBLM on safety consciousness and further curbing the negative effect on subordinate safety behaviors. In summary, this study proposes the following hypotheses.

**Hypothesis** **5a** **(H5a).** *Chinese traditionality moderates the mediating effect of emotional exhaustion between the LBLM and miners’ safety behavior. The higher the level of Chinese traditionality, the weaker the mediating effect of miners’ emotional exhaustion.*

**Hypothesis** **5b** **(H5b).** *Chinese traditionality moderates the mediating effect of safety consciousness between the LBLM and miners’ safety behavior. The higher the level of Chinese traditionality, the stronger the mediating effect of miners’ safety consciousness.*

## 3. Materials and Methods

### 3.1. Participants and Procedure

The participants in this study came from 14 coal mining companies in Liaoning, Inner Mongolia, and Shanxi, China. This survey was approved by mine managers, and participation was completely voluntary and participants had the right to withdraw from the survey at any stage. The survey collected data by distributing questionnaires on-site during group training or pre-shift meetings for miners, and a brief introduction was given before the survey to emphasize the voluntary and anonymous nature of this survey, and the formal survey was conducted with the consent of the participants. During the survey, the researchers answered questions from the participants about the content of the questionnaire. The survey was conducted from March 2022 to June 2022, and a total of 583 questionnaires were distributed and collected on the spot. Taking into account the study specifics and referring to the experience of previous studies [26], this study used the following three criteria to screen for invalid questionnaires: (a) Questionnaire content with more than 10% missing entries (N = 49); (b) more than 80% of the questionnaire items had consistent options (N = 81); (c) two or more options appeared multiple times for the same question item (N = 31). A total of 161 invalid questionnaires were excluded, and 422 valid questionnaires were recovered, with a valid recovery rate of 72.4%.

The final sample consisted of 402 males (95.3%) and 20 females (4.7%), of which 80.8% subjects were married. The subjects’ education was mainly concentrated in high school or college (80.1%). The age of the subjects was mainly distributed between 26 and 45 years old (67.3%), and the years of working in the current enterprise were mainly 3–10 years (59.4%). The demographic information of the final sample is shown in Table 1.

### 3.2. Measures

The scales used in this study were all published in authoritative journals and tested in several empirical studies in the Chinese context. In addition, a back-translation procedure was followed to translate English into simplified Chinese. All scales were 5-point Likert scales, ranging from strongly disagree (1) to strongly agree (5).

#### 3.2.1. Leader Bottom-Line Mentality

Leader bottom-line mentality was assessed by adapting a four-items scale from Greenbaum et al. [21]. A sample item is “My leader treats the bottom line as more important than anything else”. The Cronbach’α of this scale was 0.887 in the present study.

#### 3.2.2. Emotional Exhaustion

Emotional exhaustion was assessed using a six-items scale developed by Wharton [67]. A sample item is “I dread getting up in the morning and having to face another day on the job”. The Cronbach’α of this scale was 0.894 in the present study.

#### 3.2.3. Safety Consciousness

Safety consciousness was assessed using a seven-items scale developed by Barling et al. [56]. A sample item is “I am well aware of the safety risks involved in my job”. The Cronbach’α of this scale was 0.880 in the present study.

#### 3.2.4. Chinese Traditionality

Chinese traditionality was assessed using a seven-items scale developed by Farh et al. [62]. A sample item is “The best way to avoid mistakes is to follow the instructions of senior persons”. The Cronbach’α of this scale was 0.862 in the present study.

#### 3.2.5. Safety Behavior

Safety behavior was assessed using a six-items scale developed by Neal and Griffin [9], which contains two dimensions: safety compliance and safety participation. Sample items include “I use all the necessary safety equipment to do my job” and “I promote the safety program within the organization”, respectively. The Pearson correlation coefficient of the two dimensions was 0.79, indicating a high correlation. Referring to the practice of previous studies, this study takes the sum of the scores of the two dimensions as the score of safety behavior [68,69]. The Cronbach’α of safety behavior was 0.893 in the present study.

#### 3.2.6. Control Variables

Referring to previous studies, this study used gender, age, marital status, work experience in current enterprise, and education as control variables [14,24].

### 3.3. Analytic Strategy

SPSS 26.0 and Mplus 8.3 were used for data analysis in this study. First, the study used SPSS 26.0 to calculate Cronbach’s α for all variables to assess the internal consistency reliability of the variables, and Confirmatory Factor Analysis (CFA) was conducted using Mplus 8.3, the AVE and CR were calculated for each variable to assess the discriminant validity and convergent validity of the variables. Secondly, this study used SPSS 26.0 and Mplus 8.3 to perform descriptive statistical analysis for each variable and conduct common method bias test. Third, we constructed a structural equation model (SEM) using Mplus 8.3 and tested the parallel mediating path with 5000 bootstrapping. Finally, given the drawbacks of regression analysis in testing for moderated mediating effects and based on the recommendations of Cheung and Lau [70] and the testing procedure of Cheung et al. [71], this study used the LMS (Latent Moderated Structural Equations) to test the moderated mediation model. The results of the analysis were further validated by using 5000 bootstrapping.

## 4. Results

### 4.1. Preliminary Analyses

According to Table 2, the Cronbach’α of the variables in this study were all greater than 0.7, indicating good internal consistency reliability of the variables, and all variables’ AVE > 0.5, CR > 0.7, indicating good convergent validity within the variables in this study. In addition, the Confirmatory Factor Analysis (CFA) results showed (as shown in Table 3) that the five-factor model had the best fit (χ^2^/df = 1.527, CFI = 0.972, TLI = 0.969, RMSEA = 0.035, SRMR = 0.038), and the one-factor model had a poor fit, indicating good discriminant validity for the data.

Table 4 shows the results of the descriptive statistical analysis for each variable, including the mean, standard deviation, and Pearson correlation coefficient. The square root of the AVE for the variables in the diagonal of the table were greater than the correlation coefficients, further indicating good discriminant validity among the variables. Furthermore, as expected from the study, LBLM was significantly and negatively correlated with miners’ safety behavior (r = −0.60, *p* < 0.01), and H1 was initially tested.

### 4.2. Common Method Bias Test

Although the study used process control and other means to reduce the common method bias, the problem of common method bias may still exist because the data in this study were obtained from miners’ self-reports. Given this, this study took the following approach to test: first, the Harman single factor test results showed that the first factor explained 34.96%, below the recommended threshold of 40%, so there was no significant common method bias [72]. However, according to the suggestion of Podsakoff et al. [73], the Harman singer factor test may be inadequate in detecting common method bias. Therefore, the Unmeasured Latent Method Construct (ULMC) was further used in this study to detect common method bias in this study [74]. The model was slightly improved by adding the common method factor to the five-factor model (χ^2^/df = 1.421, CFI = 0.980, TLI = 0.975, RMSEA = 0.032, SRMR = 0.030), but by comparing the two model fits, Δχ^2^/df = 2.75, which is lower than the critical value of 3.84 proposed by Williams et al. [75]. In summary, there is no serious common method bias in this study.

### 4.3. The Mediation Effect Test

This study constructed a structural equation model using Mplus 8.3 and tested the mediating effect of emotional exhaustion and safety consciousness between LBLM and safety behavior based on 5000 bootstrapping as suggested by Williams and Mackinnon [76]. As shown in Table 5, LBLM significantly and negatively affected employees’ safety behaviors (effect = −0.132, *p* < 0.01, 95% CI = [−0.202, −0.052]), and H1 was tested. LBLM significantly and negatively affected miner’s safety behavior through emotional exhaustion (effect = −0.119, *p* < 0.001, 95% CI = [−0.164, −0.080]), and H2 was verified. In addition, the indirect effect of LBLM through safety consciousness on miner’s safety behavior was also negative and significant (effect = −0.137, *p* < 0.001, 95% CI = [−0.179, −0.093]), and H3 was tested.

### 4.4. The Moderated Mediation Effect Test

Currently, the most popular method for testing moderated mediating effects is regression analysis, but the regression coefficients and confidence intervals generated by regression analysis may be biased [70]. Given this, this study used Latent Moderated Structural Equations (LMS) to test the moderating effect of Chinese traditionality according to the suggestion of Cheung et al. [71].

Since the conventional model fit indices for the LMS model were not available from Mplus, a baseline model (model 0) without the latent interaction term was constructed first, and determine the fit of the LMS model (model 1) by comparing the Akaike information criterion (AIC) of the two models and performing a log-likelihood ratio test [77,78]. The baseline model fit was better (χ^2^/df = 1.539, CFI = 0.961, TLI = 0.957, RMSEA = 0.036, SRMR = 0.050), and by comparing the fit of the two models (as shown in Table 6), AIC (Model 0) − AIC (Model 1) > 0, Δdf = 1, D = −2 × (Loglikelihood h0 (Model 0) − Loglikelihood h0 (Model 1)) = 7.002, and the log-likelihood ratio test is significant (*p* < 0.01) according to the results of the chi-square distribution, indicating that the baseline model has a significant loss in fit compared to the LMS model. In summary, the LMS model fits well and can be used for the next step of the moderated mediation test.

**Figure 2:**
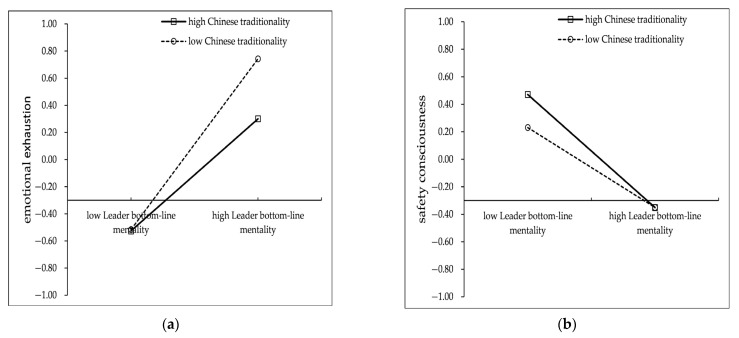
Moderating effect of Chinese traditionality. (**a**) The moderation of Chinese traditionality on emotional exhaustion. (**b**) The moderation of Chinese traditionality on safety consciousness.

First, the moderating effect of Chinese traditionality on the effect of LBLM on miners’ emotional exhaustion and safety consciousness was examined, and the results are shown in Table 7. Based on 5000 bootstrapping, the interaction between LBLM and Chinese traditionality significantly negatively affected miner’s emotional exhaustion (effect = −0.100, *p* < 0.01, 95% CI = [−0.165, −0.035]). When miners’ Chinese traditionality was high (Mean + SD), the positive effect of LBLM on miners’ emotional exhaustion was 0.382, *p* < 0.001, 95% CI = [0.286, 0.481], and when miners’ Chinese traditionality was low (Mean − SD), the positive effect of LBLM on miners’ emotional exhaustion was 0.583, *p* < 0.001, 95% CI = [0.469, 0.693], and the difference between the two cases was −0.201, *p* < 0.01, 95% CI = [−0.331, −0.069], indicating that Chinese traditionality significantly and negatively moderated the relationship between LBLM and miners’ emotional exhaustion, H4a was tested. In addition, the interaction between LBLM and Chinese traditionality also significantly and negatively affected employees’ safety consciousness (effect = −0.056, *p* < 0.01, 95% CI = [−0.113, −0.005]). When miners’ Chinese traditionality was high (Mean + SD), the negative effect of LBLM on safety consciousness was −0.380, *p* < 0.001, 95% CI = [−0.457, −0.310], −0.269, *p* < 0.001, 95% CI = [−0.350, −0.188] when miners’ Chinese traditionality was low (Mean − SD), and the difference was −0.111, *p* < 0.05, 95% CI = [−0.226, −0.009], indicating that Chinese traditionality significantly and negatively moderated the relationship between LBLM and miners’ safety consciousness, H4b was verified. To further demonstrate the moderating effect of Chinese traditionality, the study conducted a simple slope test, as shown in Figure 2.

Then, also based on 5000 bootstrapping, the moderating effect of Chinese traditionality on the mediating effect of miners’ emotional exhaustion and safety consciousness was then examined using the LMS. In the mediation pathway of miners’ emotional exhaustion, the index of moderated mediation 1 (IndexMM1) was 0.025, *p* < 0.01, 95% CI = [0.009, 0.047]. The mediating effect of miners’ emotional exhaustion was −0.096, *p* < 0.001, 95% CI = [−0.140, −0.064] when miners’ Chinese traditionality was high (Mean + SD) and −0.146, *p* < 0.001, 95% CI = [−0.204, −0.098] when miners’ Chinese traditionality was low (Mean − SD), and the difference was 0.050, *p* < 0.01, 95% CI = [0.018, 0.095], indicating that Chinese traditionality significantly moderated the mediating effect of miners’ emotional exhaustion, H5a was tested. In the miners’ safety consciousness mediated pathway, the IndexMM2 of Chinese traditionality was −0.023, *p* < 0.05, 95% CI = [−0.052, −0.003]. The mediating effect of miners’ safety consciousness was −0.160, *p* < 0.001, 95% CI = [−0.225, −0.107] when miners’ Chinese traditionality was high (Mean + SD) and −0.113, *p* < 0.001, 95% CI = [−0.168, −0.073] when miners’ Chinese traditionality was low (Mean − SD), the difference was −0.047, *p* < 0.05, 95% CI = [−0.105, −0.006], indicating that Chinese traditionality significantly moderated the mediating relationship of miners’ safety consciousness, and H5b was tested. The test results of all the research hypotheses are shown in Table 8.

## 5. Discussion

### 5.1. Theoretical Implications

This study makes the following major theoretical contributions. First, this research enriches the literature on LBLM by introducing it to the field of safety-critical workplaces. Most of the emerging research on LBLM has focused on the effects of LBLM in general organizational contexts, while this study examines the effects of LBLM on miners’ safety behaviors, responding to previous calls to explore the effects of LBLM in different industries [27]. Furthermore, the results of previous studies suggested that LBLM is a double-edged sword in general organizational contexts, as it can improve organizational performance in the short term but is detrimental to long-term organizational development [22]. This study argues that the positive effects of the LBLM will be lost when the bottom-line outcome is contrary to the expected outcome, such that the LBLM will myopically view creativity as a hindrance to achieving organizational performance and inhibit team creativity [42]. The results of this study further support the idea that LBLM inhibits employee safety performance when the leader’s bottom-line outcome (i.e., financial interest) conflicts with the primary goal of the coal mine (i.e., safety).

As the distributor of information and allocator of resources within the organization, leaders have a key role in influencing employees’ safety behaviors [11,12]. However, most previous studies have focused on the effects of constructive leadership on employee safety behaviors, and insufficient research has been conducted on destructive leadership [12]. As a practical but short-sighted management method, LBLM is also prevalent in an increasingly competitive business environment [22,79]. Given the harmful and prevalent nature of LBLM, this study further enriches the literature on safety behaviors by identifying LBLM as a hindering factor affecting employee safety behaviors.

Third, based on dual-system theory, this study introduces emotional exhaustion and safety consciousness as mediators to explore the dual path mechanism of LBLM to influence employees. In previous studies, many scholars have explained the formation mechanism of employee safety behavior based on the dual-system theory [12,33,36]. However, while automatic system 1 plays an important role in predicting safety behavior, it has been relatively neglected compared to controlled system 2 [37], especially in the leadership literature in safety-critical workplaces. In addition, we must focus on the core difference between LBLM and other negative leadership popular in safety workplaces (e.g., abusive management). LBLM not only drains employees’ physical and mental resources like abusive supervision, but also goes further to subconsciously erode employees’ proper perceptions of safety. Therefore, this study proposes that LBLM not only leads to emotional exhaustion (disabling employees’ cognitive system 2) but also reduces employees’ safety consciousness (contaminating employees’ cognitive system 1), which in turn leads to a decrease in employees’ safety behavior. This not only enriches the influence mechanism of the LBLM, but also further broadens the explanatory power of dual-system theory for safety behavior and the application scenarios in the safety field.

Finally, the study introduces Chinese traditionality as the boundary of the LBLM to influence subordinates’ safety behavior. Previous research has explored many individual differences that enhance or diminish the effects of LBLM, but attention to cultural value orientations remains insufficient [60,79]. Especially for workplaces like coal mines, which are relatively closed and have high power distance, individual cultural value orientations may play a more significant role [27]. On the one hand, the moderating role of Chinese traditionality answers the question: why do employees internalize rather than reject the attitudes and perceptions of their leaders when the leader is concerned only with bottom-line outcomes and not with employee well-being? Individuals with high Chinese traditionality tend to obey and internalize the leader’s attitudes when confronted with the leader’s bottom-line mentality, even if the leader’s communicated perceptions are incorrect. On the other hand, the findings suggest that LBLM hurts the safety behaviors of employees with different cultural orientations, only the path of its effects may differ. For employees with low Chinese traditionality, employees experience more emotional exhaustion and a slight decrease in safety consciousness, so the main reason for their reduced safety behavior is the inability of system 2 to function adequately or properly. For individuals with high traditionality, on the other hand, employees actively or passively internalize the misconceptions of the leader’s bottom-line mentality and experience less emotional exhaustion, in this case, the decline in employee safety behavior is mainly due to the contamination of cognitive system 1. This is further evidence of the absolute dysfunction of the LBLM in the safe workplace.

### 5.2. Practical Implications

The results of this study have some practical contributions to managers and organizations. First, our findings demonstrate that the LBLM of the avid pursuit of financial interests is a completely dysfunctional leadership style in a high-risk industry such as coal mining enterprises. While shaping a poor safety climate, the bottom-line mentality also leads to a vicious cycle within the organization. Specifically, the profit-oriented mindset of the LBLM makes employees more inclined to focus on job performance at the expense of safety performance, and employees who value safety either gradually assimilate into this narrow mindset or choose to leave the organization [61]. The remaining employees are generally weak in safety consciousness and lack the willingness and ability to engage in safety behaviors. Moreover, when most individuals in a group do not engage in safety behaviors, hidden risks within the organization will accumulate over time [9]. Therefore, LBLM is highly undesirable for managers of coal mines, and leaders should never neglect the long-term stability of the mine for the sake of immediate, petty profits. For coal mining enterprises, the most important resource that leaves the mine every day is not coal but miners [80,81]. Therefore, coal mining enterprises need to use human resource management strategies to identify LBLM within the organization and keep them out. However, LBLM is insidious and it is often difficult for coal mining enterprises to identify this negative leadership. Therefore, coal mining enterprises also need to broaden employee voice channels, pay close attention to employee perceptions of leaders, identify leaders within the organization who hold a bottom-line mentality, and improve the organization’s safety management.

Second, our findings suggest that emotional exhaustion and safety consciousness are pathways through which the LBLM affects subordinates’ safety behaviors, which gives organizations ideas on how to mitigate the negative effects of the LBLM and improve safety behaviors. While China has always placed a high priority on coal mine safety, we have to admit that there are still many short-sighted and profit-minded leaders who uphold or are building this dangerous management ideology. Therefore, coal mining companies also need to improve miners’ safety consciousness and pay attention to their physical and mental state from an employee-centered perspective, so that miners have adequate coping ability in the face of the LBLM. Specifically, on the one hand, coal mining companies should strictly implement the organization’s internal safety management norms, impose reasonable penalties for violations of safety regulations, effectively carry out safety training, and raise employees’ safety consciousness so that they know exactly what safety is and how to respond safely to accidents. On the other hand, organizations should pay attention to employees’ well-being and psychological demands, and try to prevent employees from falling into a negative state of overload or emotional exhaustion, so that employees can have enough resources to engage in safety behavior and make rational judgments when facing safety decision-making.

Third, the results of the study show that Chinese traditionality moderates the relationship between LBLM and subordinates’ cognition and safety behavior. For coal mining companies, although they should focus on both physical and mental health and safety consciousness of employees, differentiated management for employees with different personalities can more effectively help employees cope with the LBLM. For employees with high traditionality, it is easier to be inculcated with wrong safety concepts by the LBLM, the organization should focus on strengthening safety training for these employees, and when necessary, tougher management methods can be used to improve the training effect. For employees with low traditionality, they are more likely to fall into emotional exhaustion in the face of the LBLM, and the organization should pay attention to the mental health status of such employees and provide timely detachment and guidance.

In conclusion, this study concludes that LBLM should be considered a negative leadership style not only in coal mines but also in other organizations. Although many recent studies have begun to explore the positive aspects of LBLM, some researchers have chosen to use the term “myopic focus” to describe this management style even though they believe that LBLM as a practical leadership is beneficial to the financial performance of organizations [22]. As Wolfe [39] argues, an organization is a multivalent system that requires the coordination of multiple values and needs. Therefore, this study argues that regardless of what the bottom-line reflects (profit, safety, ethics, etc.), leaders with a high bottom-line mentality are dysfunctional because they focus too narrowly on their bottom-line goals to the exclusion of the rest of the organization’s values and needs [27]. Regardless of the type of organization, managers should be cautious about using this leadership style, even if it may lead to temporary gains.

### 5.3. Limitations and Future Research Directions

First, for the sensitivity of the questions covered in this study’s questionnaire, in order to reduce employees’ concerns about information exposure, this study used subordinates’ self-reports to obtain data, which is more common in the safety research literature [24], but also inevitably suffers from common method bias. Therefore, we minimized common method bias by emphasizing procedural controls such as anonymity, on-the-spot questionnaire collection, and answering subjects’ questions about the questionnaire. The results of the data analysis also indicated that there was no serious problem of common method bias in this study. However, in order to further reduce common method bias, we also suggest that future studies broaden the data source channels by using supervisors’ or colleagues’ evaluations and objective accident data as measures of miners’ safety behavior. In addition, measuring miners’ safety consciousness through questionnaires may be influenced by social desirability, so future studies may measure miners’ attentional bias toward safety as the measure of miners’ cognitive system 1 [12].

Second, based on dual-system theory, this study proposes that the LBLM would inhibit employees’ safety behaviors by affecting subordinates’ emotional exhaustion (cognitive system 2) and safety consciousness (cognitive system 1) in turn. However, according to dual-system theory, the normal functioning of cognitive system 2 is often based on cognitive system 1 as well [45]. Therefore, the two cognitive systems are not completely independent and may influence each other. On the one hand, based on the hedonic principle, it is the human instinct to approach pleasure and avoid pain, and when miners have sufficient resources for self-control, they will refuse to internalize the misconceptions instilled by the LBLM; therefore, the miner’s emotional exhaustion may affect their safety consciousness. On the other hand, the leader’s managerial attitude of neglecting safety is an important cause of miners’ emotional resource depletion, but when miners’ safety consciousness is low, they may have less negative emotions about the leader’s neglect of safety, i.e., miners’ safety consciousness may also influence their emotional state. Future research could further explore the interaction between miners’ safety consciousness and emotional exhaustion and the impact on miners’ safety behavior through longitudinal study (e.g., cross-lagged model).

In addition, this study explores the effect of LBLM on employee safety behavior only at the employee’s cognitive level, which may not be sufficient to fully explain the negative effect of LBLM on safety behavior. In fact, LBLM may disregard operating environments with safety hazards in order to improve financial interests and force miners to put in extra time and effort to achieve performance goals. In addition, leaders may also reduce safety inputs to save costs, which increases the objective danger of the working environment. These operations may cross miners’ cognitive status and directly act on their safety behaviors, but it is obviously an important reason for the decline in the safety performance of coal mines. It is suggested that future research could further select objective indicators such as injuries and accidents in coal mines to measure the negative impact of LBLM on coal mine safety performance.

Greenbaum et al. [42] suggested that future research could focus on whether bottom-line mentality exhibits constructive outcomes when bottom-line outcomes reflect indicators other than financial performance (e.g., safety, ethics, etc.). However, this study does not focus on the safety-centered LBLM, but rather on the traditional LBLM. In fact, it has been suggested that employees with safety as a bottom-line outcome exhibit higher levels of safety performance [82], and there is no doubt that a safety-specific LBLM is likely to lead to higher safety performance in organizations. However, this does not mean that we should shift directly from pursuing financial bottom-line results to pursuing safety bottom-line, which is also undesirable as an overkill. In recent years, with the gradual adjustment of China’s energy structure and the increase in the requirements of coal mines, the survival pressure on coal mine companies has continued to increase [5,83]. Therefore, in order to maintain the normal operation of the organization, a more important principle for coal mining companies should be to coordinate the development of multiple values and needs of the organization while ensuring that safety is at the top of the list. In addition, this study also recommends that future researchers further focus on whether LBLM exhibits constructive outcomes when it reflects different bottom-line outcomes and whether LBLM is nonlinearly related to bottom-line outcomes over time.

## 6. Conclusions

Based on cognitive dual-system theory, this study developed a theoretical framework for the influence of LBLM on miners’ safety behavior and provided empirical data on the relationship between LBLM and safety behavior. The findings suggest that LBLM inhibits miners’ safety behaviors by influencing their emotional exhaustion and safety consciousness. When miners’ Chinese traditionality is high, the mediating effect of emotional exhaustion is weakened, while the mediating effect of safety consciousness is strengthened. This study introduces LBLM into the safety domain, which not only expands the literature related to LBLM and safety behaviors, but also reminds coal mining companies to pay attention not only to miners’ physical and mental states, but also to their correct safety cognition, providing practical insights into how coal mining companies can reduce the negative effects of LBLM and improve miners’ safety behaviors.

## Figures and Tables

**Table 1:** Final participant demographics (N = 422).

Variables		Number	Percent (%)
Gender	Male	402	95.3
	Female	20	4.7
Age	18–25 years	69	16.4
	26–35 years	140	33.2
	36–45 years	144	34.1
	46–55 years	69	16.4
Marital	Unmarried	81	19.2
	Married	341	80.8
Education	Junior high school and below	43	10.2
	High school	141	33.4
	Technical school	202	47.9
	Undergraduate or above	36	8.5
Work experience	0–2 years	86	20.4
	3–5 years	128	30.3
	6–10 years	123	29.1
	11 years and over	85	20.1

**Table 2:** Reliability and convergent validity (N = 422).

Variables	Cronbach’α	AVE	CR
LBLM	0.887	0.6662	0.8883
EH	0.894	0.5853	0.8939
SC	0.880	0.5169	0.8817
CT	0.862	0.5564	0.8623
SB	0.893	0.5857	0.8944

Note: LBLM, leader bottom-line mentality; EH, emotional exhaustion; SC, safety consciousness; CT, Chinese traditionality; SB, safety behavior.

**Table 3:** Confirmatory factor analysis for testing structure validity (N = 422).

Model	χ^2^	df	χ^2^/df	CFI	TLI	RMSEA	SRMR
Five-Factor Model	519.195	340	1.527	0.972	0.969	0.035	0.038
Four-Factor Model	1010.459	344	2.937	0.897	0.887	0.068	0.059
Three-Factor Model	1737.656	347	5.008	0.785	0.766	0.097	0.082
two-Factor Model	2608.119	349	7.473	0.651	0.622	0.124	0.116
Single-Factor Model	2993.611	350	8.553	0.592	0.559	0.134	0.120

Note: Five-Factor Model: LBLM, EH, SC, SB, CT; Four-Factor Model: LBLM, EH, SC + SB, CT; Three-Factor Model: LBLM, EH + SC + SB, CT; two-Factor Model: LBLM, EH + SC + SB + CT; Single-Factor Model: LBLM + EH + SC + SB + CT.

**Table 4:** Descriptive statistics and correlations among variables (N = 422).

Variables	Mean	SD	1	2	3	4	5	6	7	8	9	10
1. Gender	0.95	0.21	-									
2. Age	2.50	0.95	0.00	-								
3. Marital	0.81	0.40	0.06	0.52 **	-							
4. Education	2.55	0.79	−0.03	0.02	−0.08	-						
5. WE	2.49	1.03	0.02	0.80 **	0.48 **	0.06	-					
6. LBLM	3.09	1.00	−0.06	0.06	−0.03	0.00	0.06	(0.82)				
7. EH	2.76	0.83	−0.02	0.11 *	−0.06	0.06	0.06	0.53**	(0.77)			
8. SC	3.83	0.68	0.12 *	−0.15 **	−0.01	−0.07	−0.10 *	−0.54 **	−0.36 **	(0.72)		
9. CT	3.52	0.85	0.07	0.08	0.21 **	−0.11 *	0.03	0.00	−0.12 *	0.09	(0.75)	
10. SB	3.92	0.72	0.06	−0.17 **	0.04	−0.04	−0.13 **	−0.60 **	−0.58 **	0.59 **	0.10 *	(0.77)

Note: * *p* < 0.05, ** *p* < 0.01; WE, work experience; Gender: 0 = female, 1 = male. Age: 1 = 18–25 years, 2 = 26–35 years, 3 = 36–45 years, 4 = 46–45 years. Marital: unmarried = 0, married = 1. Education: Junior high school and below = 1, High school = 2, Technical school = 3, Undergraduate or above = 4. Work experience in the current enterprise: 1 = 0–2 years, 2 = 3–5 years, 3 = 6–10 years, 4 = 11 years and over; square root of AVE are in the parentheses on the diagonal.

**Table 5:** Direct effect and indirect effect results (N = 422).

Unstandardized Effect	Estimate	S.E.	*p*-Value	95% CI
Direct effect				
LBLM → EH	0.476	0.043	0.000	[0.407, 0.560]
LBLM → SC	−0.329	0.028	0.000	[−0.390, −0.276]
LBLM → SB	−0.132	0.042	0.002	[−0.202, −0.052]
EH → SB	−0.249	0.044	0.000	[−0.324, −0.165]
SC → SB	0.417	0.072	0.000	[0.303, 0.562]
Indirect effect				
LBLM → EH → SB	−0.119	0.021	0.000	[−0.164, −0.080]
MED2 → SC → SB	−0.137	0.025	0.000	[−0.179, −0.093]

**Table 6:** Model comparison.

Model	Free Parameters	Loglikelihood h0	AIC
baseline model (model 0)	97	−13,311.357	26,816.714
LMS model (model 1)	98	−13,307.856	26,811.712

**Table 7:** Moderating effect and moderated mediating effect results (N = 422).

Unstandardized Effect	Estimate	S.E.	*p*-Value	95% CI
Moderating effect				
LBLM × CT → EH	−0.100	0.036	0.005	[−0.165, −0.035]
LBLM → EH(Mean + SD)	0.382	0.050	0.000	[0.286, 0.481]
LBLM → EH(Mean − SD)	0.583	0.058	0.000	[0.469, 0.693]
Difference	−0.201	0.068	0.003	[−0.331, −0.069]
LBLM × CT → SC	−0.056	0.025	0.025	[−0.113, −0.005]
LBLM → SC(Mean + SD)	−0.380	0.041	0.000	[−0.457, −0.310]
LBLM → SC(Mean − SD)	−0.269	0.039	0.000	[−0.350, −0.188]
Difference	−0.111	0.054	0.038	[−0.226, −0.009]
Moderated mediating effect				
IndexMM1	0.025	0.010	0.010	[0.009, 0.047]
LBLM → EH → SB(Mean + SD)	−0.096	0.019	0.000	[−0.140, −0.064]
LBLM → EH → SB(Mean − SD)	−0.146	0.026	0.000	[−0.204, −0.098]
Difference	0.050	0.019	0.010	[0.018, 0.095]
IndexMM2	−0.023	0.011	0.032	[−0.052, −0.003]
LBLM → SC → SB(Mean + SD)	−0.160	0.026	0.000	[−0.225, −0.107]
LBLM → SC → SB(Mean − SD)	−0.113	0.021	0.000	[−0.168, −0.073]
Difference	−0.047	0.022	0.032	[−0.105, −0.006]

**Table 8:** Summary of hypothesis testing results.

Hypothesis Content	Results
H1: LBLM has a negative effect on miners’ safety behavior.	support
H2: Emotional exhaustion mediates the negative effect of LBLM on miners’ safety behavior.	support
H3: Safety consciousness mediates the negative influence of LBLM on miners’ safety behavior.	support
H4a: Chinese traditionality moderates the positive relationship between LBLM and miners’ emotional exhaustion.	support
H4b: Chinese traditionality moderates the negative relationship between LBLM and miners’ safety consciousness.	support
H5a: Chinese traditionality moderates the mediating effect of emotional exhaustion between the LBLM and miners’ safety behavior.	support
H5b: Chinese traditionality moderates the mediating effect of safety consciousness between the LBLM and miners’ safety behavior.	support

## Data Availability

Data are available from the authors upon reasonable request.

## References

[1] Zhang Y., Shao W., Zhang M., Li H., Yin S., Xu Y. (2016). Analysis 320 coal mine accidents using structural equation modeling with unsafe conditions of the rules and regulations as exogenous variables. Accid. Anal. Prev..

[2] Fu G., Xie X., Jia Q., Tong W., Ge Y. (2020). Accidents analysis and prevention of coal and gas outburst: Understanding human errors in accidents. Process Saf. Environ. Prot..

[3] Zhang J., Xu K., Reniers G., You G. (2020). Statistical analysis the characteristics of extraordinarily severe coal mine accidents (ESCMAs) in China from 1950 to 2018. Process Saf. Environ. Prot..

[4] Qiu Z., Liu Q., Li X., Zhang J., Zhang Y. (2021). Construction and analysis of a coal mine accident causation network based on text mining. Process Saf. Environ. Prot..

[5] Kong B., Cao Z., Sun T., Qi C., Zhang Y. (2022). Safety hazards in coal mines of Guizhou China during 2011–2020. Saf. Sci..

[6] Liu Q., Li X., Hassall M. (2021). Regulatory regime on coal Mine Safety in China and Australia: Comparative analysis and overall findings. Resour. Policy.

[7] Kumar P., Gupta S., Gunda Y.R. (2020). Estimation of human error rate in underground coal mines through retrospective analysis of mining accident reports and some error reduction strategies. Saf. Sci..

[8] Ye X., Ren S., Chadee D., Wang Z. (2020). ‘The canary in the coal mine’: A multi-level analysis of the role of hope in managing safety performance of underground miners. J. Vocat. Behav..

[9] Neal A., Griffin M.A. (2006). A study of the lagged relationships among safety climate, safety motivation, safety behavior, and accidents at the individual and group levels. J. Appl. Psychol..

[10] Zhang S., Shi X., Wu C. (2017). Measuring the effects of external factor on leadership safety behavior: Case study of mine enterprises in China. Saf. Sci..

[11] Salancik G.R., Pfeffer J. (1978). A social information processing approach to job attitudes and task design. Adm. Sci. Q..

[12] Yuan X., Xu Y., Li Y. (2020). Resource Depletion Perspective on the Link Between Abusive Supervision and Safety Behaviors. J. Bus. Ethics.

[13] Basahel A.M. (2021). Safety Leadership, Safety Attitudes, Safety Knowledge and Motivation toward Safety-Related Behaviors in Electrical Substation Construction Projects. Int. J. Environ. Res. Public. Health.

[14] Zheng J., Gou X., Griffin M.A., Goh Y.M., Xia N. (2022). Temporal leadership, attentiveness, and safety behaviors: The moderating roles of abusive supervision and safety consciousness. Saf. Sci..

[15] Ali M., Aziz S., Pham T.N., Babalola M.T., Usman M. (2020). A positive human health perspective on how spiritual leadership weaves its influence on employee safety performance: The role of harmonious safety passion. Saf. Sci..

[16] Rose K., Shuck B., Twyford D., Bergman M. (2015). Skunked: An Integrative Review Exploring the Consequences of the Dysfunctional Leader and Implications for Those Employees Who Work for Them. Hum. Resour. Dev. Rev..

[17] Yang L.Q., Zheng X., Liu X., Lu C.Q., Schaubroeck J.M. (2020). Abusive supervision, thwarted belongingness, and workplace safety: A group engagement perspective. J. Appl. Psychol..

[18] Lyubykh Z., Turner N., Hershcovis M.S., Deng C. (2022). A meta-analysis of leadership and workplace safety: Examining relative importance, contextual contingencies, and methodological moderators. J. Appl. Psychol..

[19] Feng Y., Xing Z. (2021). Research on impacts of destructive leadership on safety performance based on conditional process analysis. China Saf. Sci. J..

[20] Zhang J., Zeng Y., Reniers G., Liu J. (2022). Analysis of the Interaction Mechanism of the Risk Factors of Gas Explosions in Chinese Underground Coal Mines. Int. J. Environ. Res. Public. Health.

[21] Greenbaum R.L., Mawritz M.B., Eissa G. (2012). Bottom-line mentality as an antecedent of social undermining and the moderating roles of core self-evaluations and conscientiousness. J. Appl. Psychol..

[22] Babalola M.T., Greenbaum R.L., Amarnani R.K., Shoss M.K., Deng Y., Garba O.A., Guo L. (2020). A business frame perspective on why perceptions of top management’s bottom-line mentality result in employees’ good and bad behaviors. Pers. Psychol..

[23] Quade M.J., Wan M., Carlson D.S., Kacmar K.M., Greenbaum R.L. (2021). Beyond the Bottom Line: Don’t Forget to Consider the Role of the Family. J. Manag..

[24] Fruhen L.S., Andrei D.M., Griffin M.A. (2022). Leaders as motivators and meaning makers: How perceived leader behaviors and leader safety commitment attributions shape employees’ safety behaviors. Saf. Sci..

[25] Lin Y., Yang M., Quade M.J., Chen W. (2022). Is the bottom line reached? An exploration of supervisor bottom-line mentality, team performance avoidance goal orientation and team performance. Hum. Relat..

[26] Xia N., Xie Q., Hu X., Wang X., Meng H. (2020). A dual perspective on risk perception and its effect on safety behavior: A moderated mediation model of safety motivation, and supervisor’s and coworkers’ safety climate. Accid. Anal. Prev..

[27] Babalola M.T., Mawritz M.B., Greenbaum R.L., Ren S., Garba O.A. (2021). Whatever It Takes: How and When Supervisor Bottom-Line Mentality Motivates Employee Contributions in the Workplace. J. Manag..

[28] Evans J.S. (2003). In two minds: Dual-process accounts of reasoning. Trends Cogn. Sci..

[29] Babalola M.T., Ren S., Ogbonnaya C., Riisla K., Soetan G.T., Gok K. (2022). Thriving at work but insomniac at home: Understanding the relationship between supervisor bottom-line mentality and employee functioning. Hum. Relat..

[30] Zhang Y., Zhang H., Xie J., Yang X. (2021). Coping with Supervisor Bottom-Line Mentality: The Mediating role of Job Insecurity and the Moderating Role of Supervisory Power. Curr. Psychol..

[31] Wu T.-J., Li J.-M., Wu Y.J. (2022). Employees’ job insecurity perception and unsafe behaviours in human–machine collaboration. Manag. Decis..

[32] Wan W.H., Zhang D.N., Liu X.Y., Jiang K.J. (2021). How do Chinese employees respond to leader bottom-line mentality? A conservation of resources perspective. Soc. Behav. Pers..

[33] Zhang J., Wu C. (2014). The influence of dispositional mindfulness on safety behaviors: A dual process perspective. Accid. Anal. Prev..

[34] Zhao H., Liu W. (2020). Managerial coaching and subordinates’ workplace well-being: A moderated mediation study. Hum. Resour. Manag. J..

[35] Farh J.-L., Hackett R.D., Liang J. (2007). Individual-level cultural values as moderators of perceived organizational support-employee outcome relationships in China: Comparing the effects of power distance and traditionality. Acad. Manag. J..

[36] Chen M., Chen L., Yan X.M., Yu Z., Fang Y.Y., Yu Y.Q. (2018). Investigating the nonlinear effect of ego depletion on safety compliance: The moderating role of rumination. J. Saf. Res..

[37] Xu Y., Li Y., Ding W., Lu F. (2014). Controlled versus automatic processes: Which is dominant to safety? The moderating effect of inhibitory control. PLoS ONE.

[38] Griffin M.A., Neal A. (2000). Perceptions of safety at work: A framework for linking safety climate to safety performance, knowledge, and motivation. J. Occup. Health Psychol..

[39] Wolfe D.M. (1988). Is there integrity in the bottom line: Managing obstacles to executive integrity. Executive Integrity: The Search for High Human Values in Organizational Life.

[40] Quade M.J., McLarty B.D., Bonner J.M. (2019). The influence of supervisor bottom-line mentality and employee bottom-line mentality on leader-member exchange and subsequent employee performance. Hum. Relat..

[41] Tai K., Lee K., Kim E., Johnson T.D., Wang W., Duffy M.K., Kim S. (2021). Gender, bottom-line mentality, and workplace mistreatment: The roles of gender norm violation and team gender composition. J. Appl. Psychol..

[42] Greenbaum R.L., Bonner J.M., Mawritz M.B., Butts M.M., Smith M.B. (2020). It is all about the bottom line: Group bottom-line mentality, psychological safety, and group creativity. J. Organ. Behav..

[43] Gagné M., Deci E.L. (2005). Self-determination theory and work motivation. J. Organ. Behav..

[44] Irshad M., Majeed M., Khattak S.A. (2021). The Combined Effect of Safety Specific Transformational Leadership and Safety Consciousness on Psychological Well-Being of Healthcare Workers. Front. Psychol..

[45] Evans J.S. (2008). Dual-processing accounts of reasoning, judgment, and social cognition. Annu. Rev. Psychol..

[46] Maslach C., Schaufeli W.B., Leiter M.P. (2001). Job Burnout. Annu. Rev. Psychol..

[47] Li F., Jiang L., Yao X., Li Y. (2013). Job demands, job resources and safety outcomes: The roles of emotional exhaustion and safety compliance. Accid. Anal. Prev..

[48] Elsaied M. (2022). Exploitative leadership and organizational cynicism: The mediating role of emotional exhaustion. Leadersh. Organ. Dev. J..

[49] Kong D.T., Ho V.T., Garg S. (2020). Employee and Coworker Idiosyncratic Deals: Implications for Emotional Exhaustion and Deviant Behaviors. J. Bus. Ethics.

[50] Chen Y., Li S. (2020). Relationship Between Workplace Ostracism and Unsafe Behaviors: The Mediating Effect of Psychological Detachment and Emotional Exhaustion. Psychol. Rep..

[51] Hao Q., Wei K., Zhang B. (2022). How to attenuate the effects of abusive supervision on knowledge hiding: The neutralizing roles of coworker support and individual characteristics. J. Knowl. Manag..

[52] Wu H., Qiu S., Dooley L.M., Ma C. (2020). The Relationship between Challenge and Hindrance Stressors and Emotional Exhaustion: The Moderating Role of Perceived Servant Leadership. Int. J. Environ. Res. Public. Health.

[53] Kamran K., Azam A., Atif M.M. (2022). Supervisor Bottom-Line Mentality, Performance Pressure, and Workplace Cheating: Moderating Role of Negative Reciprocity. Front. Psychol..

[54] Farasat M., Azam A., Hassan H. (2021). Supervisor bottom-line mentality, workaholism, and workplace cheating behavior: The moderating effect of employee entitlement. Ethics Behav..

[55] Lin W., Koopmann J., Wang M. (2020). How Does Workplace Helping Behavior Step Up or Slack Off? Integrating Enrichment-Based and Depletion-Based Perspectives. J. Manag..

[56] Barling J., Loughlin C., Kelloway E.K. (2002). Development and test of a model linking safety-specific transformational leadership and occupational safety. J. Appl. Psychol..

[57] de Koster R.B.M., Stam D., Balk B.M. (2011). Accidents happen: The influence of safety-specific transformational leadership, safety consciousness, and hazard reducing systems on warehouse accidents. J. Oper. Manag..

[58] Seibert S.A. (2014). Safety consciousness: Assignments that expand focus beyond the bedside. Nurse Educ. Today.

[59] Khan N., Ahmad I., Ilyas M. (2018). Impact of Ethical Leadership on Organizational Safety Performance: The Mediating Role of Safety Culture and Safety Consciousness. Ethics Behav..

[60] Zhang Y., He B., Huang Q.H., Xie J. (2020). Effects of supervisor bottom-line mentality on subordinate unethical pro-organizational behavior. J. Manag. Psychol..

[61] Mesdaghinia S., Rawat A., Nadavulakere S. (2019). Why Moral Followers Quit: Examining the Role of Leader Bottom-Line Mentality and Unethical Pro-Leader Behavior. J. Bus. Ethics.

[62] Farh J.-L., Earley P.C., Lin S.-C. (1997). Impetus for action: A cultural analysis of justice and organizational citizenship behavior in Chinese society. Adm. Sci. Q..

[63] Li S.-L., Huo Y., Long L.-R. (2017). Chinese Traditionality Matters: Effects of Differentiated Empowering Leadership on Followers’ Trust in Leaders and Work Outcomes. J. Bus. Ethics.

[64] Yang C., Chen Y., Zhao Roy X., Mattila A.S. (2020). Unfolding deconstructive effects of negative shocks on psychological contract violation, organizational cynicism, and turnover intention. Int. J. Hosp. Manag..

[65] Cheng K., Guo L., Luo J. (2021). The more you exploit, the more expedient I will be: A moral disengagement and Chinese traditionality examination of exploitative leadership and employee expediency. Asia Pacific J. Manag..

[66] Wang J., Kim T.-Y. (2013). Proactive socialization behavior in China: The mediating role of perceived insider status and the moderating role of supervisors’ traditionality. J. Organ. Behav..

[67] Wharton A.S. (1993). The Affective Consequences of Service Work:Managing Emotions on the Job. Work Occup..

[68] Yu M., Qin W., Li J. (2022). The influence of psychosocial safety climate on miners’ safety behavior: A cross-level research. Saf. Sci..

[69] Xu Q., Wu Y., Wang M., Liu B., Jiang J., You X., Ji M. (2022). The relationship between sense of calling and safety behavior among airline pilots: The role of harmonious safety passion and safety climate. Saf. Sci..

[70] Cheung G.W., Lau R.S. (2017). Accuracy of Parameter Estimates and Confidence Intervals in Moderated Mediation Models. Organ. Res. Methods.

[71] Cheung G.W., Cooper-Thomas H.D., Lau R.S., Wang L.C. (2021). Testing Moderation in Business and Psychological Studies with Latent Moderated Structural Equations. J. Bus. Psychol..

[72] Harman H.H. (1976). Modern Factor Analysis.

[73] Podsakoff P.M., MacKenzie S.B., Lee J.Y., Podsakoff N.P. (2003). Common method biases in behavioral research: A critical review of the literature and recommended remedies. J. Appl. Psychol..

[74] Hulland J., Baumgartner H., Smith K.M. (2018). Marketing survey research best practices: Evidence and recommendations from a review of JAMS articles. J. Acad. Mark. Sci..

[75] Williams L.J., Hartman N., Cavazotte F. (2010). Method Variance and Marker Variables: A Review and Comprehensive CFA Marker Technique. Organ. Res. Methods.

[76] Williams J., Mackinnon D.P. (2008). Resampling and Distribution of the Product Methods for Testing Indirect Effects in Complex Models. Struct Equ Modeling.

[77] Maslowsky J., Jager J., Hemken D. (2015). Estimating and interpreting latent variable interactions: A tutorial for applying the latent moderated structural equations method. Int. J. Behav. Dev..

[78] Sardeshmukh S.R., Vandenberg R.J. (2017). Integrating Moderation and Mediation: A Structural Equation Modeling Approach. Organ. Res. Methods.

[79] Babalola M.T., Jordan S.L., Ren S., Ogbonnaya C., Hochwarter W.A., Soetan G.T. (2022). How and When Perceptions of Top Management Bottom-Line Mentality Inhibit Supervisors’ Servant Leadership Behavior. J. Manag..

[80] Saleh J.H., Cummings A.M. (2011). Safety in the mining industry and the unfinished legacy of mining accidents: Safety levers and defense-in-depth for addressing mining hazards. Saf. Sci..

[81] Yang X., Krul K., Sims D. (2022). Uncovering coal mining accident coverups: An alternative perspective on China’s new safety narrative. Saf. Sci..

[82] Sun S., Ren H. Employees’ Bottom Line Mentality and Safety Behaviors: The Moderating Role of Safety Climate. Proceedings of the 5th International Workshop on Advances in Energy Science and Environment Engineering.

[83] Yi T., Dong Y., Li J. (2021). Influence of job insecurity on coal miners’ safety performance: The role of emotional exhaustion. Chinese Manag. Stud..

